# Complex Visceral Coupling During Central Sleep Apnea in Cats

**DOI:** 10.3389/fnins.2020.00568

**Published:** 2020-06-17

**Authors:** Alexandra V. Limanskaya, Irina I. Busygina, Ekaterina V. Levichkina, Ivan N. Pigarev

**Affiliations:** ^1^Institute for Information Transmission Problems (Kharkevich Institute), Russian Academy of Sciences, Moscow, Russia; ^2^Department of Higher Nervous Activity, Faculty of Biology, Lomonosov Moscow State University, Moscow, Russia; ^3^Pavlov Institute of Physiology, Russian Academy of Sciences, Saint Petersburg, Russia; ^4^Department of Optometry and Vision Sciences, The University of Melbourne, Parkville, VIC, Australia

**Keywords:** central sleep apnea, heart rate, respiration, stomach motility, duodenal motility, visceral theory of sleep

## Abstract

Central sleep apnea is a sudden arrest of breathing during sleep caused by the central commands to the thoracoabdominal muscles. It is a widespread phenomenon in both healthy and diseased people, as well as in some animals. However, there is an ongoing debate whether it can be considered as a pathological deviation of the respiratory function or an adaptive mechanism of an unclear function. We performed chronic recordings from six behaving cats over multiple sleep/wake cycles, which included electroencephalogram, ECG, eye movements, air flow, and thoracic respiratory muscle movements, and in four cats combined that with the registration of myoelectric activity of the stomach and the duodenum. In these experiments, we observed frequent central cessations of breathing (for 5–13 s) during sleep. Each of the sleep apnea episodes was accompanied by a stereotypical complex of somatic and visceral effects. The heart rate increased 3–5 s before the respiration arrest and strongly decreased during the absence of respiration. The myoelectric activity of the stomach and the duodenum also often demonstrated a strong suppression during the apnea episodes. The general composition of the visceral effects was stable during all periods of observation (up to 3 years in one cat). We hypothesize that the stereotypic coupling of activities in various visceral systems during episodes of central sleep apnea most likely reflects a complex adaptive behavior rather than an isolated respiratory pathology and discuss the probable function of this phenomenon.

## Introduction

Sleep-related breathing disorders, including central sleep apnea in humans, currently are at the focus of attention of many sleep studies. Generally, central apnea manifests as a short absence or reduction of breathing during sleep (9–14-s in length) caused by the cessation or the attenuation of the central respiratory drive from the breathing control center to the thoracoabdominal muscles. This phenomenon, or rather a group of phenomena, has a complex manifestation. Apnea can occur as an isolated disorder or as one of the numerous symptoms accompanying other illnesses (e.g., congenital heart failure and obstructive sleep apnea), or it can even be an intrinsic feature of the normal breathing system of a healthy organism, for instance, in high-altitude conditions or in early infancy (Hernandez and Patil, 2016; Baillieul et al., 2019).

Although recent advances in sleep research provided valuable descriptions of the various aspects of this phenomenon and of its manifestation types (Eckert et al., 2007; Javaheri et al., 2017), there is still no agreement regarding the possible functional role of central sleep apnea. The noticed link between central sleep apnea and the malfunction of the cardio-respiratory system led some researchers to an assumption that central apnea represents a pathological phenomenon related to a failure in breathing control and therefore requires specific treatment, while others proposed that it may have an important adaptive purpose (see e.g., Gay, 2008; Malhotra et al., 2008; Cowie, 2017). Similar effects with common features to those described in human subjects were also observed in kittens (McGinty et al., 1979), as well as in rodent models (e.g., Sato et al., 1990; Nakamura et al., 2003; Davis and O’Donnell, 2013).

During a series of studies not focused on apnea but devoted to the exploration of cortical representations of the visceral system in wakefulness and in sleep (Pigarev et al., 2013, 2016), which required a prolonged collection of somnographic data over multiple sleep/wake cycles, we, to our surprise, realized that central apnea is a frequent event of sleep in cats. Moreover, we noted a correlation between changes of the multiple parameters of visceral functioning surrounding the apnea episodes. In this paper, we describe these patterns of co-occurrence of visceral disturbances and discuss their possible adaptive function. These results were partly presented in abstract form (Limanskaya, 2019).

## Materials and Methods

In this study, we retrospectively analyzed recordings obtained in the course of four different previous projects, and therefore we had an opportunity to analyze data from six healthy adult cats.

All experiments with these cats were performed using our modification of the painless head fixation approach (Noda et al., 1971; Pigarev et al., 2009), necessary to record stable polysomnography from animals during a long period of time, up to 8 h a day. The recordings used for further analysis covered periods of both wakefulness and sleep. The number of experimental sessions analyzed in a single cat varied from 20 to 78.

Surgery and day-to-day treatment of the animals were carried out in accordance with the ethical principles for the maintenance and use of animals in neuroscience research (Zimmermann, 1987), the NIH Guidelines for the Care and Use of Animals, and the Declaration of Helsinki on Ethical Principles for Medical Research. Current Russian laws do not bind scientific institutes to have special ethic committees; the assessment of research proposals is instead conducted by the institutional scientific councils in the course of their discussion concerning providing financial support to a particular study. According to the rules of the foundations that distribute grants for scientific research in Russia, ethics evaluation is done by the council of reviewers prior to making a decision regarding the financial support of a particular study. Both councils are guided by the recommendations of the above-mentioned documents.

The preparation of the animals for the experiments included an acclimatization of each animal to the laboratory environment and either one (in two cats) or two separate surgeries (in four cats), with recovery intervals after each of them. The general approach to chronic studies conducted in behaving animals such as cats is to purchase an animal of a reasonably young age (∼1 year) and acclimatize it to the laboratory environment and to the investigators involved in the experiments. This “shaping” process was based on positive reinforcement techniques and usually took a couple of weeks before any surgery was performed.

Both surgeries were conducted under deep anesthesia (premedication with xylazine, 0.15 ml/kg; for the main anesthesia, we used zoletil, 6 mg/kg, for the first injection, and additional doses of 5 mg periodically with intervals of about 20 min to keep the appropriate level of animal sedation.

During the first surgery, a pre-fabricated halo frame for subsequent painless head fixation during the recording session was attached to the skull. The frame was manufactured using a thin steel wire as a base, with acrylic dental cement as filling and cover, and attached to the skull with eight 2.5-mm surgical-grade steel screws. The skin and soft tissues were removed from the top of the cranium (from the area inside the frame), and the surface of the skull was covered with a thin layer of acrylic dental cement [for a detailed description, see Pigarev et al. (2009)]. At this stage, two electrodes manufactured from thin (0.5 mm) Elgiloy wire for electroencephalogram (EEG) monitoring were implanted epidurally over the frontal and the occipital cortices.

After a complete recovery (at least 4 weeks), the animal was trained to stay for a prolonged time with its head fixed to ensure stable recordings over sleep/wake cycles. The duration of this training depended on the individual behavior of an animal. Usually within a couple of weeks, an animal gets acclimatized to head fixation and begins sleeping with its head fixed. After that, the second surgery can be performed. During the second surgery, intramural bipolar electrodes were implanted, allowing the recording of myoelectrical activity from the stomach and the duodenum. Intramural electrodes were implanted into the walls of these organs using the method proposed by Papasova and Milenov (1965) and Papasova et al. (1966b). The details of this procedure were previously described in Pigarev et al. (2013) as well. The same type of anesthesia was used for this procedure.

For the ECG recording, we used one lead from the stomach wall, and the second was from the ground screw inserted in the bone at the top of the skull. Since the recordings necessary to investigate central sleep apnea were mainly done in sleep, when the animal does not move, mostly there were no motion artifacts, and the QRS complexes in ECG and the R maxima of the QRS were automatically identified by Spike 2 built-in algorithms for spike sorting by shape. In rare cases when intense muscle jerks occurred during rapid eye movement (REM) sleep and resulted in artifacts, the corresponding correction was done after a visual inspection. Moments of R pick maxima were used to calculate the heart rate mean over time. For this procedure, we also used Spike 2 built-in algorithm, which replaced each event with Gaussian kernel (exponential time constant of 3 s).

The experiments were conducted during daytime in a diffusely illuminated room, with permanent video monitoring of the animal.

All standard polysomnographic parameters were recorded: cortical EEG, ECG, respiratory movements (using a thoracoabdominal belt with a piezoelectric sensor), air flow (with thermo-sensors placed in front of the nose), eye movements, and opening/closing of the eyelids (with an infrared oculometer). In addition to that, in four animals, we recorded the myoelectrical activity of the stomach and the duodenum.

All these signals were amplified with NeuroBioLab amplifiers stored on a hard drive using LabChart system (ADInstrument, Australia) and analyzed offline using LabChart and Spike 2 (CED, GB) programs. The ECG and duodenum myoelectric signals were recorded with 1 kHz sampling rate. For all other parameters, the sampling rate was 200 Hz.

## Data Analysis

Polysomnograms and video recordings were visually examined for the presence of respiratory cessation in sleep (apnea and cessation of movements of the thoracoabdominal muscles longer than 5 s), and the durations of respiratory arrests were estimated.

The separation of the states of vigilance was based on a visual inspection of the polysomnograms and on video recordings. As signs of slow wave (SW) sleep, we used the increase of delta wave amplitudes in EEG, slowing of respiration, general decrease of the animal’s motor activity, slow gaze drifts replacing saccadic eye movements, and closing of the eyelids. After selecting the intervals of SW sleep and wakefulness using these criteria, we performed a quantitative comparison of the power spectral density between these two conditions.

The EEG of the assumed SW sleep and wakefulness periods was broken into 10-s intervals, and the EEG spectra were calculated (Chronux data analysis toolbox for Matlab^1^). We analyzed the EEG spectra within frequency ranges known to depend on the state of vigilance: delta, sleep spindle, and gamma ranges. We found that, in all recordings, the delta and the spindle range power spectral density values in EEG were significantly higher in SW sleep while power in gamma range was always higher in wakefulness (Wilcoxon rank sum test *p* < 0.001). These criteria commonly characterize sleep—wake differences (Contreras and Steriade, 1996; Destexhe et al., 1999). Periods of REM sleep could be easily determined as occurring just after intervals of SW sleep desynchronization of the general EEG—the presence of eye and lid movements and very typical jerks of facial muscles as observed on video.

**FIGURE 1:**
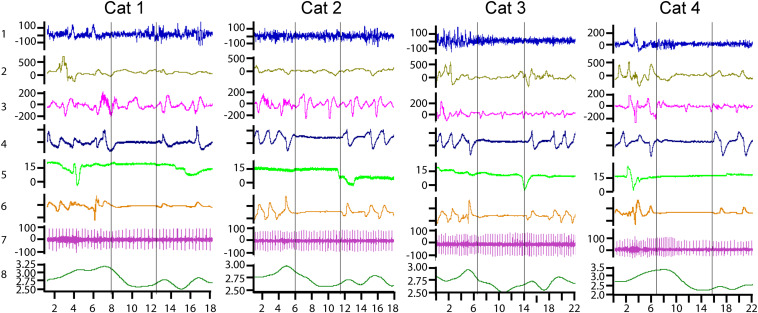
Examples of central sleep apnea episodes recorded in four cats. Channel 1—cortical electroencephalograph (EEG), μV; channel 2—myoelectric activity of the stomach, μV; channel 3—myoelectric activity of the duodenum, μV; channel 4—nasal air flow in arbitrary units; channel 5—vertical eye movements, degrees of the visual angle; channel 6—movements of the thoracoabdominal respiratory muscles in arbitrary units; channel 7—ECG in μV; and channel 8—heart rate curve, heart beats per second. The horizontal axis shows the time scale in seconds.

The myoelectrical activities of the stomach and the duodenum were analyzed only for estimation of the reduction of their motility-related features during apnea episodes. For stomach activity, this manifested as an absence or a reduction of high-amplitude myoelectrical waves during periods of respiratory cessations (Figure 1, channel 2). For the duodenum, this manifested as the absence of high-frequency spike potentials, as can be seen in Figure 1 (channel 3). For a detailed description of the relationships between the myoelectrical activity of the stomach and the duodenum and their motility, see Papasova et al. (1966a), Costa and Furness (1982), Sarna (1989), and Martinez-de-Juan et al. (2000).

## Results

The presented results were obtained from a retrospective analysis of the polysomnograms recorded in six cats, which were used in four different projects devoted to the investigation of various aspects of sleep in chronic experiments.

In all six animals, episodes of central sleep apnea were found in most of the analyzed experiments.

Figure 1 presents typical examples of central sleep apnea episodes as recorded in the four cats. All of them were registered in the transitional period from REM to SW sleep. Two vertical lines mark the borders of each apnea episode. Respiration arrest is seen in channels 4 and 6. Channel 4 represents nasal airflow and channel 6 represents the movements of the thoracic cage.

The heart rate increased 3–5 s before the cessation of breathing in all the observed sleep apnea episodes in all six cats. By the onset of the apnea episodes, the heart rate always decreased and remained at a low level until the first breath. During the apnea episodes, the intervals between two sequential heartbeats could sometimes be twice longer than during periods before the respiration arrest (e.g., Figure 1, cat 4, channel 7).

An example of heart palpitations can be seen in the ECG (channel 7) as well as in the heart rate curve (channel 8). During the recorded sleep apnea episodes (e.g., 115 of 293 in one cat), even the ones that happened during REM sleep, the eyes and the eyelids remained practically immobile (channel 5).

Channels 2 and 3 demonstrate the myoelectrical activity recorded from the walls of the stomach and the duodenum correspondingly. It is seen that, at the time of apnea onset, the myoelectric stomach activity (channel 2) gradually disappeared. The periodic myoelectrical duodenal activity (channel 3) persists. However, these periodic slow waves reflect the electrical activity of the enteric nervous system and not the peristaltic intestinal movements. Intestinal motility is more related to the higher-frequency spike potentials superimposed on these slow waves (Papasova et al., 1966a; Costa and Furness, 1982; Sarna, 1989; Martinez-de-Juan et al., 2000). Such spike potentials are seen in Figure 1, just before the apnea episodes in cats 1 and 2. However, the spike potentials were usually absent during respiratory arrests. Thus, it seems that duodenal and stomach motility might be decreased or absent during central sleep apnea.

In two cats (cats 1 and 2 in Figure 1), the results of all 78 daily experiment sessions (from 2 to 8 h in length) conducted during 1 year (67 on the first and 11 on the second animal) were analyzed for the presence of central sleep apnea episodes. These episodes were detected in 59 out of 78 experiments on the first cat (293 apnea episodes detected) and in 10 out of 11 experiments on the second cat (71 episodes). In these two cats, the cardio-respiratory activity and the activity of the stomach and the duodenum were recorded. For one of these two cats, with 78 daily experiments and 293 apnea episodes, the descriptive statistics of the various aspects of central sleep apnea episodes distribution over sleep–wake cycle are presented in Table 1. The common features of central apnea episodes demonstrated in Figure 1 were first identified and examined in these two animals.

**TABLE 1:** Description of central sleep apnea episodes recorded in one cat during 1 year.

	**Slow wave sleep (SWS)/rapid eye movement (REM) transition**	**REM sleep**	**Slow wave sleep**	**Total**
Number of apnea episodes	183 (62.4%)	63 (21.5%)	47 (16%)	293 (100%)
Mean duration of apnea episode (s)	9.8 ± 2.26	9 ± 2.58	9.9 ± 2.49	9.7 ± 2.37
Number of episodes with the absence of eye movements	105 (57%)	37 (59%)	33 (70%)	175 (58%)
Number of apnea episodes with the reduction in myoelectric activity of the stomach and/or the intestine	115 (63%)	33 (52%)	34 (72%)	182 (62%)
Mean (±SEM) interval between episodes (min)	13.1 ± 10.4	15.97 ± 14.4	16 ± 9.3	14.3 ± 10.1

In two cats (3 and 4 in Figure 1), the recorded visceral parameters also included heart rate, respiration, and activity of the stomach and the duodenum. In the remaining two cats (5 and 6), the visceral parameters included only the activity of the heart and respiration. In these four cats (3, 4, 5, and 6), we analyzed only quasi randomly selected experiments (20 in each cat) that were uniformly distributed across the periods from the beginning to the end of these studies (1–3 years). The goal of this analysis was to determine whether central sleep apnea episodes and their typical pattern were present in all animals during the entire intervals of these studies.

Table 1 summarizes the numbers and the durations of apnea episodes per sleep state and the frequencies of the effects co-occurring with the apnea episodes for one cat’s recordings performed during 1 year.

The table shows that the largest number of respiratory arrests was observed during the transition periods between SW and REM sleep (the direction of the transition period was not taken into account and the data were pooled together). The durations of apnea episodes obviously did not differ for the different phases of sleep.

We noticed only one episode of central sleep apnea during the transition from wakefulness to SW sleep.

## Discussion

Central apnea episodes during sleep were observed in all six cats during natural sleep. This corresponds to the numerous descriptions of these effects given in previous animal studies on kittens, rats, and mice (McGinty et al., 1979; Sato et al., 1990; Nakamura et al., 2003; Davis and O’Donnell, 2013). The novel element of this study was the observation of cardio-respiratory coupling in all observed apnea episodes. In addition, in a substantial fraction of registered sleep apnea episodes, we also noted stabilization of the eyes and eyelid movements, and the changes of stomach and intestinal myoelectric activity suggested a decrease in their motility.

The co-occurrence of the changes in heart rate, the myoelectric activity of the duodenum and the stomach, and ocular motions with the breathing arrests, the stereotypical pattern of these visceral changes in sleep, and the absence of such organized changes in wakefulness seem indicative of the existence of some coordinated central neural program that controls central apneas specifically during sleep. A central apnea episode therefore represents a complex effect involving multiple visceral systems rather than an isolated one, happening only in the respiratory system.

An obvious limitation of this study was in the number of simultaneously recorded visceral parameters. It would be important to enlarge this list in the future in order to see whether other visceral systems also can be engaged in this coordinated process. The other omission is the inability to correlate these events to the activity of the brain structures closely involved in the regulation of these parameters and thus to find the origin of such stereotypically orchestrated changes of the visceral parameters.

In a cat observed for 3 years, the pattern of apnea episodes remained constant during this prolonged period. The age of the cats used in our study varied from 1 to 5 years, and episodes of central apnea were noted at all ages. These animals passed veterinary examinations and there were no indications on any pathological deviations in their health.

The recorded episodes of central sleep apnea had a characteristic pattern of coordinated changes occurring in several visceral systems. Namely, cardiac changes preceded the ones in breathing, and changes in breathing were frequently accompanied by stereotypical changes of gastrointestinal activity. What could be the functional role of such coordinated complex which exists only in sleep?

Our previous studies demonstrated that, during sleep, multiple cortical areas, which processed various exteroceptive signals during wakefulness, switch to processing of the interoceptive information [see, e.g., Pigarev (1994), Pigarev et al. (2013)]. Thus, the cerebral cortex becomes substantially visceral during sleep. We proposed that, during sleep, the cerebral cortex is engaged in a diagnostic of the visceral state of an organism and the restoration of the detected defects in various visceral systems. For that reason, the cerebral cortex receives and processes interoceptive signals during sleep (Pigarev, 2014; Pigarev and Pigareva, 2014, 2015).

Note that the observed complex activity during central apnea always starts from an increase of the heart rate. Together with simultaneous deep respiratory movements, the increased heart rate provides all tissues, first of all the brain, with a sufficient amount of oxygen. Only after that, respiration stops and the heart rate decreases, and the gastrointestinal motility slows down as well. We hypothesize that such simultaneous visceral “dying down” is necessary to synchronize the analysis of incoming visceral information in the brain, the information that normally represents organs of different rhythmicity. This mechanism can presumably achieve a similar result to the one existing in the visual system, namely, the saccadic suppression, when information processing is severely diminished during saccadic eye movements in order to achieve stability of visual perception (Benedetto and Morrone, 2017). A similar idea is used in stroboscopic methods of investigation of moving objects, e.g., in MRI investigations of the heart’s structure, when scanning moments are synchronized with a particular phase of the heart cycle. In the case of central apnea, the heart strongly reduces its frequency in order to increase the time interval when it would be possible to get a reading of information from stable heart and respiratory systems. Other visceral systems, if necessary, join this process of total stabilization. The stabilization of the eyes also indicates a reduction of activity in the oculomotor cortical areas which, in line with the visceral theory of sleep (Pigarev, 2014), are also involved in the processing of visceral information during sleep and potentially capable of sending viscero-motor commands.

Our observations suggest that the selective elimination of sleep apnea might lead to health problems, potentially starting with the cardio-respiratory function but not limited to it. A strong increase of the heart rate before respiratory arrest potentially allows testing this assumption in a relatively simple experiment. Using a permanent automatic monitoring of the heart rate, it seems possible to detect increasing heart rate frequency and deliver some alarming stimulation to interrupt sleep and, consequently, respiratory arrest. A similar approach to heart rate monitoring is used in some automatic devices designed for vagus nerve stimulation because the heart rate was found to be increased before epileptic seizures in many cases. In epileptic patients, modern vagal stimulation devices detect increasing heart rate during sleep and switch on a stimulation of the vagus nerve to prevent the development of convulsive activity [see, e.g., Dibue-Adjei et al. (2019)]. Elimination of the entire visceral complexes accompanying the apnea episodes would allow assessing the possible consequences of this action for the health of the animals. However, considering that frequent awakening might have a negative effect by itself, it would be necessary to have a group of control animals that get the same alarming signals irrespective of the presence of apnea in their sleep. This approach is similar to the one successfully used in the disk-over-water experimental devices for a sleep deprivation research in the classical experiments conducted in the laboratory of Allan Rechtschaffen (Bergmann et al., 1989).

Within the frame of the proposed functional role of central sleep apnea, the increased number of these events may indicate potential health problems related to cardio-respiratory function. Attempts to eliminate central apneas by some artificial manipulations may lead to aggravation of the existing problems. In fact, the use of adaptive servo-ventilation to eliminate the respiratory influences of central apnea was found to be harmful rather than helpful in some cases, leading to increased mortality—mainly of sudden death related to arrhythmic events (Cowie et al., 2015), and a specific adaptive role of central apnea was suggested (Cowie, 2017). The increased number of sleep apnea episodes at high altitudes in healthy subjects (Mees and de la Chaux, 2009) also fits well to the assumption of the regulatory role of central apnea. The same logic explains the increased number of central sleep apnea episodes in the case of obesity or smoking (Block et al., 1979). We suggest that, during unexpected alteration of cardio-respiratory function, the brain needs information concerning the states of various organs in order to develop a strategy for survival, and this can be realized during periods of central sleep apnea.

## Data Availability Statement

The datasets generated for this study are available on request to the corresponding author.

## Ethics Statement

Ethical review and approval was not required for the animal study because surgery and day-to-day treatment of the animals were carried out in accordance with the ethical principles for the maintenance and use of animals in neuroscience research (Zimmermann, 1987), NIH guidelines for the care and use of animals and Declaration of Helsinki on Ethical Principles for Medical Research. Current Russian lows do not bind scientific institutes to have special ethic committees; instead, assessment of the research proposals is conducted by the Institutional Scientific Councils in the course of their discussion concerning providing financial support to a particular study. In foundations, distributing grants for scientific researches, ethic evaluation is done by Council of reviewers before a decision concerning financial support of a study. Both these councils in general are guided by the recommendations of the above-mentioned documents.

## Author Contributions

AL and IP contributed conception and design of the study, and collected the data. IB, AL, and IP performed operations. All authors analyzed the data, contributed to manuscript revision, and read and approved the submitted version. AL, IP, and EL wrote the first draft of the manuscript.

## Conflict of Interest

The authors declare that the research was conducted in the absence of any commercial or financial relationships that could be construed as a potential conflict of interest.

## Footnotes

1 http://chronux.org
